# Rifaximin Improves Visceral Hyperalgesia via TRPV1 by Modulating Intestinal Flora in the Water Avoidance Stressed Rat

**DOI:** 10.1155/2020/4078681

**Published:** 2020-07-20

**Authors:** Chang-qing Yang, Xiao-shu Guo, Ji-Li, Zi-bai Wei, Li Zhao, Gan-ting Zhao, Shu-ting Sheng

**Affiliations:** ^1^Department of Gastroenterology, Heping Hospital Affiliated to Changzhi Medical College, Changzhi, Shanxi Province, China; ^2^Department of Physiology, Changzhi Medical College, Changzhi, Shanxi Province, China

## Abstract

**Background:**

Rifaximin is effective in relieving pain symptoms with IBS patients, although the mechanisms were not clear. The aims of the research were to investigate whether the visceral hyperalgesia was alleviated by rifaximin via TRPV1 channel in rats.

**Methods:**

Rats were subjected to water avoidance stress (WAS) and were pretreated with rifaximin by oral gavage. The visceromotor response to colorectal distension was measured. The changes of TRPV1 in peripheral and central neurons of rats were detected by immunofluorescence, western blot method, and RT-PCR. Bacterial 16S ribosomal DNA in ileal contents was assessed using the Illumina MiSeq platform. The effect of intestinal flora on TRPV1 channel was observed by fecal microbiota transplantation (FMT) methods.

**Results:**

Rifaximin could relieve the visceral hyperalgesia and reduce the TRPV1 expression of neurons and ileum mucosa in rats induced by WAS. The reduced relative abundance of intestinal flora induced by WAS could be partly prevented by rifaximin. The electromyographical activities and immunoreactivity of TRPV1 in rats could be changed after FMT.

**Conclusions:**

Rifaximin could improve visceral hyperalgesia via TRPV1 channels of peripheral and central neurons by modulating intestinal flora in rats.

## 1. Introduction

Irritable bowel syndrome (IBS) is the most common functional gastrointestinal disorder that affects approximately 10-20% of the world's population [1]. Abdominal pain is the most common symptom in patients with IBS; however, the management of pain is clinically difficult and ineffective. Studies indicated that visceral hyperalgesia, increased sensation pressure threshold to rectal distention, was one of the important pathogenesis of abdominal pain in patients with IBS [2]. Rifaximin, as a broad-spectrum intestinal antibiotic, has been reported to alter intestinal bacteria and prevent visceral hyperalgesia in response to chronic psychological stress [3]. However, the exact mechanisms were unclear.

Signals from the gut to the brain can be affected by many factors from different organs. Intestinal flora were regarded as an important element in the regulation of intestinal homeostasis [2]. The brain signals from visceral afferents may be affected by various stimuli which interacted with the intestinal flora. Many studies have shown that some probiotics have an obvious effect in relieving pain symptom of patients with IBS [4].

The obvious increase of transient receptor potential vanilloid 1 (TRPV1), an receptor of pain perception, was observed in gut and primary sensory neurons from animals and patients with IBS [5]. In our previous study, we have demonstrated that TRPV1 signals were activated in visceral hyperalgesia rats induced by stress and infection [6]. Oral administration of *Lactobacillus reuteri* could reduce the capsaicin-induced TRPV1 ionic current in DRG primary culture from rats [7]. Therefore, we assessed whether rifaximin improves visceral hyperalgesia via TRPV1 channel by modulating the intestinal flora in chronic stress rats.

## 2. Materials and Methods

### 2.1. Animals

Adult male Sprague-Dawley rats were housed in ultraclean conditions using ventilated racks and in groups of 3-5 per cage. They were maintained at 22°C with an automatic 12 hour light/dark schedule with lights. The experimental procedures were approved by the Changzhi Medical College Animal Ethics Committee.

### 2.2. Water Avoidance Stress (WAS)

As previously described [3], rats were placed on a platform in the middle of a plexiglas tank filled with sterile water (25°C), and the platform was 1 cm taller than the water. They were kept on the platform for 1 hour daily for 10 days. There was no water in the tank in the sham WAS rat groups. In a separate study, rats were pretreated with rifaximin by oral gavage when they received a WAS procedure. Rats were dosed with rifaximin 150 mg/kg, twice daily, 6 hours apart (a.m. and p.m.), for 10 days.

### 2.3. Fecal Microbiota Transplant (FMT) and Measurement of the Motility of the Isolated Ileum

We investigated whether gut visceral sensitivity, TRPV1, and ileum muscle contractility were affected by the changes of intestinal flora induced by rifaximin. Before FMT, the digestive contents of the rats were cleaned by gavage with 200 *μ*L 425 g/L polyethylene glycol (PEG), four times, at 20 min intervals as already described [8]. After 4 hours, control rats received FMT by gavage with 200 *μ*L resuspended feces in PBS from donors (WAS+Rifaximin rats and WAS+saline rats) for 3 days. Further experiments began one week later.

Rats were sacrificed for cervical dislocation and then laparotomy was performed as reported earlier [9, 10]. The ileum was immediately removed and placed in a petri dish containing Tyrode's solution at room temperature. The petri dish was continuously supplied with a mixture of 95% O_2_ and 5% CO_2_. The attached mesenteries were removed with tweezer, and the contents of the enteric cavity were carefully rinsed with a syringe. The 2 cm long ileum strip was used to measure the influence of resuspended feces on the contractility of the smooth muscle in rats. One end of the strips was attached to a glass vent hook, and the other ends were attached to a tension transducer connected to BL-420F physiological recording system. Before recording the activity curve of ileum smooth muscle, the contraction of the muscle strips was adjusted to 1.5 g preload and balanced for 30 min. The contractile amplitude and frequency of ileum smooth muscle were calculated, and the average recording time of the contractile curve should be over 5 min.

### 2.4. The Visceromotor Response (VMR) to Colorectal Distension (CRD)

Visceral hyperalgesia was detected by electromyographical (EMG) measurement of VMR to CRD as described previously [11]. The rats were fasting, but not deprived of water, for 24 hours before detection. After the rats were anesthetized with ether, the balloon was wetted with paraffin oil and inserted into 2 cm from the anus. We used a concentric circular electrode positioned in the external oblique muscle near the midline 1.5 cm to record the EMG signals with BL-420F biological and functional experimental system. The main parameter settings are as follows: sweep speed 250 ms/div, sample frequency 50 Hz, sensitivity 500 *μ*V, and filter frequency 1 kHz. The electrical activities of abdominal muscle were recorded after 30 min when the rats adapted to the environment. The distension volume increased gradually in 0.1 mL steps from 0 to 0.7 mL, and each distension lasting 10 s with 5 min intervals. There were 6 rats in each group, and each expansion was carried out only once.

### 2.5. Immunofluorescence

L6S1 dorsal root ganglions (DRGs) were removed, and the slides were hydrated in PBS with 1% Triton X-100 for 2 hours and then blocked with 10% normal goat serum for 30 min at room temperature. The slides were incubated overnight at 4°C with rabbit anti-TRPV1 antibody (1 : 200, Abcam). Sections were washed and incubated with goat anti-rabbit H&L (Alexa Fluor 594) (1 : 2000, Abcam) at 37°C for 30 min. The nuclei in slides were stained with DAPI Fluoromount (Solarbio) for 5 min at room temperature. Five slices of images of per DRG of per animal under the same parameters were selected for analysis with confocal microscopy (Zeiss LSM880, Germany) at low magnification (×200 objective). The immunofluorescence activities in these digital images were processed with the Image-Pro Plus 6.0 image analysis software system (Media Cybernetics, Silver Spring, MD, USA).

### 2.6. Western Blot Analysis

The L6S1 segments of the spinal cord were quickly removed, and the protein extractions were processed. The proteins were separated with 10% SDS-polyacrylamide gel electrophoresis and transferred onto PVDF membranes. Nonspecific binding sites were blocked at room temperature with 5% nonfat milk for 1 hour. The membranes were incubated overnight with rabbit anti-TRPV1 antibody (1 : 500, Abcam) at 4°C in PBS-T containing 2% nonfat dry milk. The membranes were then incubated with secondary antibody (goat anti-rabbit H&L (HRP), 1 : 1000, Abcam) for 1 hour and developed using X-ray films by the West Dura Super signal chemiluminescence kit (Pierce, Rockford, Illinois, USA). The calculation of protein bands were normalized to *β*-actin levels.

### 2.7. RT-PCR

The RNA was extracted from ileum mucosa tissue using the RNeasy Micro Kit (Qiagen) according to the manufacturer's instructions. The concentration and integrity of RNAs were further determined by absorbance at 260 nm and the electrophoresis method. The primer sequences for quantitative reverse transcription PCR for the target gene TRPV1 and endogenous control 18S ribosomal RNA are as follows: TRPV1 (sense: 5′TGGTACTGTACTTCAGCCAACGC′3, antisense: 5′ GAACACGAGGTAGACGAACATAAA′3); 18S rRNA (sense: 5′GTAACCCGTTGAACCCCATT′3, antisense: 5′CCATCCAATCGGTAGTAGCG′3). The samples were amplified using the following thermal cycling conditions: 95°C for 3 min followed by 40 cycles of amplification at 95°C for 15 s and then 60°C for 1 min to allow for denaturation and annealing extension. After amplification, a dissociation curve was plotted against melting temperature to ensure the amplification of a single product. Comparative cycle threshold values were recorded, and the relative expression of mRNA species was then quantified in duplicate using the 2^-*ΔΔ*CT^ method using the iCycler optical system interface software (v2.0, Bio-Rad).

### 2.8. 16S rDNA Sequencing

Extraction and purification of microbial DNA from the distal ileal contents of rats were performed as instructed in the QIAamp Fast DNA Stool Mini Kit (Qiagen). DNA was then submitted to the PCR amplification of the 16S V3-V4 region of bacteria. Primer sequences are as follows: forward primer (5′-3′) ACTCCTACGGGRSGCAGCAG and reverse primer (3′-5′) GGACTACVVGGGTATCTAATC. The KAPA HiFi HotStart Ready Mix PCR kit was used to ensure the accuracy and efficiency of the PCR amplification. DNA library should have a quality inspection and quantitative analysis with Qubit. The 16S rDNA sequencing was performed and analyzed with the Illumina HiSeq PE250in Realbio Genomics Institute (Shanghai, China).

### 2.9. Statistics

Results were presented as means ± SEM. To compare the effects of stress and/or rifaximin on the VMR to CRD, 2-way repeated-measures analysis of variance (ANOVA) was used. Differences between groups in other studies were compared using 1-way ANOVA, followed by the least significant difference (LSD) test or the Student-Newman-Keuls (S-N-K) test. The differences in immunoreactive neurons after CRD between two groups were analyzed with the Student's *t* test. Statistical criteria for significant differences between groups were set at *P* < 0.05.

## 3. Results

### 3.1. Rifaximin Relieve Visceral Hyperalgesia in Rats Induced by WAS

**Figure 1:**
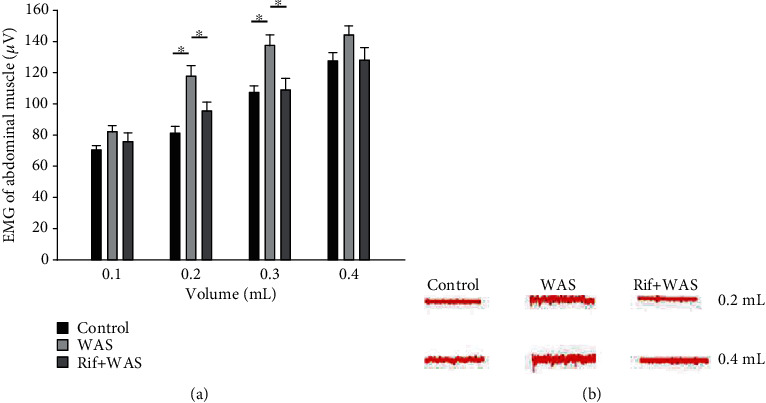
Electromyographical (EMG) activities of visceromotor response to colorectal distension (CRD). EMG activities in the external oblique muscle in response to graded CRD (distention volume: 0.1, 0.2, 0.3, and 0.4 mL) were measured in rats. Red wavy line represents a typical electromyography of corresponding groups (mean ± SEM, *n* = 6, ∗*P* < 0.05).

In response to CRD, there were no distinctions at the EMG activities of abdominal muscle when the distension volume reach 0.1 mL in rats. However, there were obvious differences when the distension volume reach 0.2 mL (control vs. WAS: 80.95 ± 4.48 vs. 117.63 ± 6.77, *P* = 0.001; WAS vs. Rif+WAS: 117.63 ± 6.77 vs. 95.49 ± 5.67, *P* = 0.015) and 0.3 mL (control vs. WAS: 107.19 ± 4.53 vs. 137.55 ± 6.49, *P* = 0.004; WAS vs. Rif+WAS: 137.55 ± 6.49 vs. 108.77 ± 7.45, *P* = 0.005). However, these differences were not found in rats when the distension volume reach 0.5 mL (control vs. WAS vs. Rif+WAS: 128.05 ± 5.30 vs. 143.97 ± 6.08 vs. 129.15 ± 7.88), 0.6 mL (control vs. WAS vs. Rif+WAS: 129.48 ± 5.47 vs. 143.46 ± 5.84 vs. 130.41 ± 7.82), and 0.7 mL (control vs. WAS vs. Rif+WAS: 130.50 ± 5.37 vs. 144.52 ± 5.41 vs. 133.97 ± 6.39) (Figure 1).

### 3.2. Rifaximin Reduce the Expression of TRPV1 in Rats Induced by WAS

**Figure 2:**
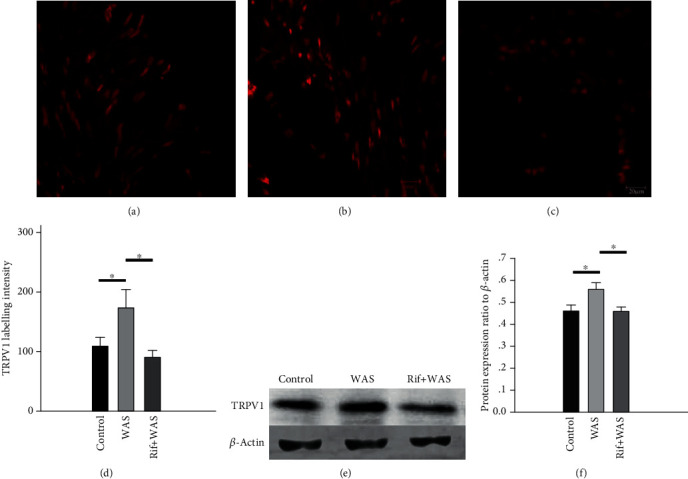
The immunoreactivity (IR) and protein expression level of TRPV1 in L6S1 dorsal root ganglions (DRGs) and in L6S1 segments of the spinal cord of rats. Representative immunofluorescence images of TRPV1 IR-positive neurons in L6S1 DRGs from the control group (a), WAS group (b), and Rif+WAS group (c). Quantification of TRPV1 IR labelling intensity (d). Representative western blot for TRPV1 in extracts from the L6S1 segment of the spinal cord (e). Relative quantification for western blot analysis (f). Data are expressed as density normalized to *β*-actin (mean ± SEM, *n* = 6, ∗*P* < 0.05).

**Figure 3:**
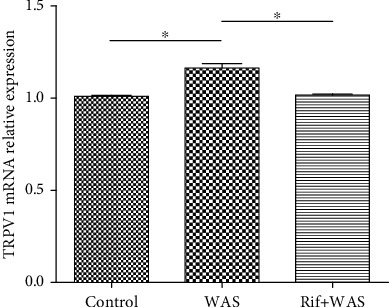
The relative levels of TRPV1 mRNA in the ileum mucosa tissue. Data was normalized to 18S ribosomal RNA and expressed using the 2^−*ΔΔ*Ct^ method (*n* = 6, ∗*P* < 0.05; ∗∗*P* < 0.01).

The immunoreactivity (IR) signals of TRPV1 were observed in L6S1 DRGs as shown in Figures 2(a)–2(c). The labelling intensity of TRPV1 IR-positive neurons in L6S1 DRGs was significantly enhanced in WAS rats than that in the control group (WAS vs. control: 173.89 ± 30.53 vs. 108.5 ± 15.02, *P* = 0.03). However, compared with the WAS group, rifaximin administration could reduce the labelling intensity of TRPV1 IR-positive neurons (Rif+WAS vs. WAS: 89.72 ± 11.70 vs. 173.89 ± 30.53, *P* = 0.006) (Figure 2(d)). The TRPV1 expressions were further identified by western blot in the L6S1 segments of the spinal cord. Compared to the control group, a significant increase in the level of TRPV1 proteins was observed in the L6S1 segments of the spinal cord in WAS rats (WAS vs. control: 0.56 ± 0.03 vs. 0.46 ± 0.03, *P* = 0.02). However, compared with the WAS group, rifaximin administration could reduce the level of TRPV1 protein (Rif+WAS vs. WAS: 0.46 ± 0.02 vs. 0.56 ± 0.03, *P* = 0.02), as shown by the quantitative densitometry evaluation of these immunoblots (Figures 2(e) and 2(f)). Compared with control (1.000 ± 0.003), a significant upregulation of TRPV1 mRNA was induced by WAS (1.163 ± 0.025, *P* < 0.001) in the ileum mucosa in rats. However, compared with the WAS group, the TRPV1 mRNA levels decreased in Rif+WAS rats (1.018 ± 0.006, *P* < 0.001) (Figure 3). This result indicated that rifaximin could alleviate the increased TRPV1 mRNA from the ileum mucosa induced by WAS.

### 3.3. Rifaximin Prevents the Changes of Special Intestinal Flora Composition in Rats Induced by WAS

**Figure 4:**
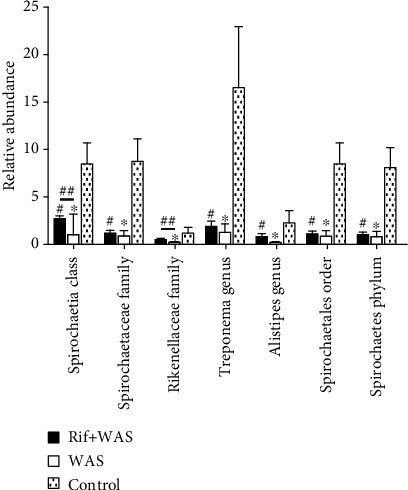
The effect of rifaximin treatment on total bacterial load and bacterial community composition in ileal contents of rats. Relative abundance of selected phenotypes, identified at class, order, phylum, family, and genus level (mean ± SEM, *n* = 6, ∗*P* < 0.05: WAS vs. control group, ^#^*P* < 0.05: Rif+WAS vs. control group, ^##^*P* < 0.05: Rif+WAS vs. WAS).

**Figure 5:**
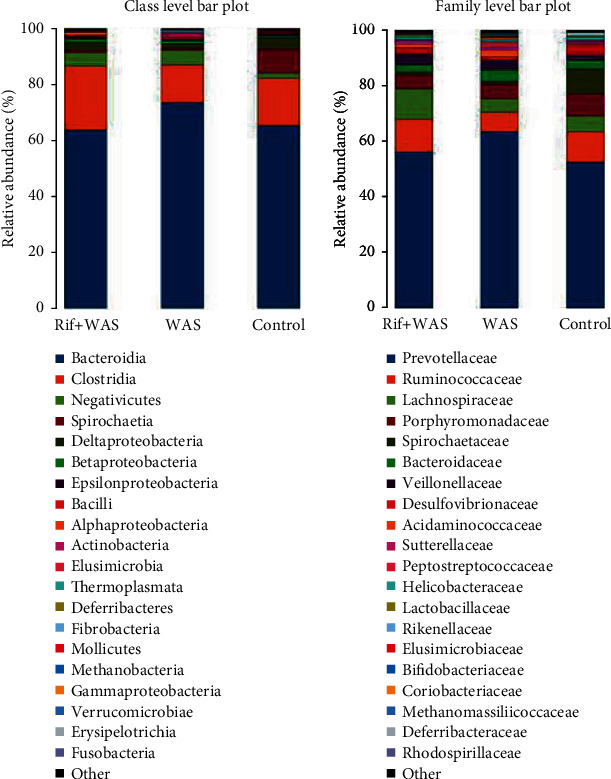
The variation in bacterial community composition in the terminal ileum at family and genus levels.

Compared to the normal control group, there was an obvious decrease in the relative abundance of intestinal flora in the WAS group or Rif+WAS group (*P* < 0.05). As the Figure 4 shows, the relative abundance of Spirochaetia, Spirochaetaceae, Rikenellaceae, Treponema, Alistipes, Spirochaetales, and Spirochaetes in different levels decreased significantly. In addition, compared with the WAS group, the relative abundance of Spirochaetia and Rikenellaceae in class and family levels increased significantly in the Rif+WAS group (*P* < 0.05) (Figures 4 and 5.

**Figure 6:**
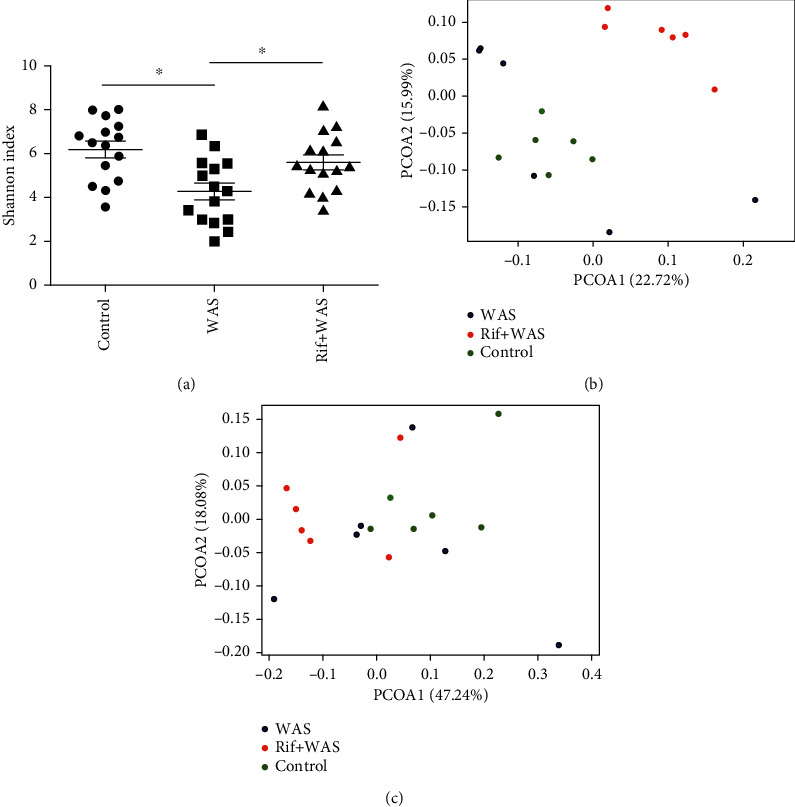
The intestinal flora profile differs in ileal contents of rats. The Shannon index of diversity (a). Principal coordinate analysis (PCoA) plots of unweighted (b) and weighted (c) UniFrac distances in which samples were coloured (mean ± SEM, *n* = 6, ∗*P* < 0.05).

By measuring alpha diversity using the Shannon index shown in Figure 6(a), compared with the control group, the Shannon index in WAS group significantly reduced (*P* < 0.001). However, compared with WAS group, the Shannon index in the Rif+WAS group significantly increased (*P* < 0.001). Beta diversity was calculated using both unweighted and weighted UniFrac phylogenetic distance matrices and visualised in PCoA plots (Figures 6(b) and 6(c)).

### 3.4. Rifaximin Improves Visceral Hyperalgesia via TRPV1 by Modulating Special Intestinal Flora

**Figure 7:**
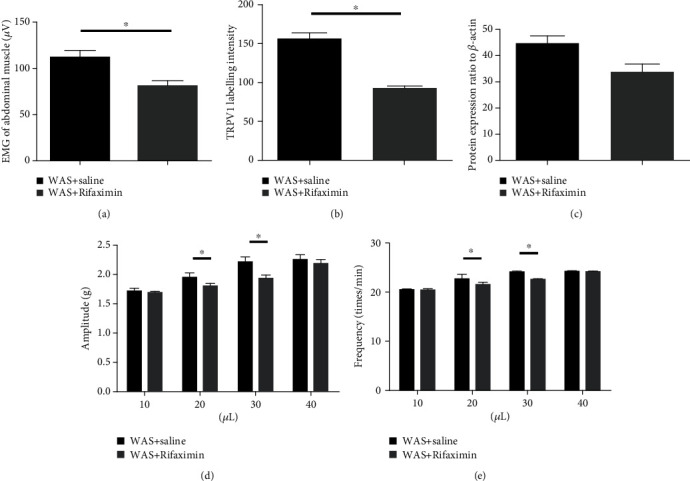
The EMG activities, changes of TRPV1, and the motility of the isolated ileum were detected in recipient mice after fecal microbiota transplantation (FMT). The EMG activities of visceromotor response to colorectal distension (a). Quantification of TRPV1 IR labelling intensity (b). Relative quantification for western blot analysis (c). Data are expressed as density normalized to *β*-actin. The contractile amplitude and frequency of isolated ileum smooth muscle of rats when it was administrated with different volumes of faecal suspension (d, e). (mean ± SEM, *n* = 6, ∗*P* < 0.05).

We used the FMT method to further evaluate whether rifaximin affects the expression of TRPV1 through the regulation of intestinal flora. The normal control rats received FMT from two different donors (WAS+Rifaximin or WAS+saline rats, respectively) by gavage. The results showed that there were significant differences on the EMG activities of abdominal muscle in rats with 0.2 mL distension volume following FMT from different rats (WAS+saline vs. WAS+Rifaximin: 112.14 ± 7.27 vs. 81.54 ± 4.79, *P* = 0.008) (Figure 7(a)). We also found that the TRPV1 IR signals were different significantly in rats following FMT from different rats (WAS+saline vs. WAS+Rifaximin: 156.72 ± 29.09 vs. 91.50 ± 15.40, *P* = 0.012) (Figure 7(b)). However, the differences of TRPV1 protein expression were not observed (Figure 7(c)).

### 3.5. Contractility of Isolated Ileum Smooth Muscle in Rats

Figures 7(d) and 7(e) indicated that resuspended feces from the donors of WAS+saline and WAS+Rifaximin could affect the contractility of the ileum smooth muscle incubated in Tyrode's solution. There were significant differences observed in contractile amplitude and frequency of the ileum smooth muscle when 20 and 30 *μ*L resuspended feces were used (*P* < 0.05).

## 4. Discussion

Intestinal flora was involved the development of visceral hyperalgesia in rats, our research indicated that antibiotic rifaximin reduced the pain threshold in water avoidance stressed rats, which were associated with the imbalance of special intestinal flora. Further researches showed that FMT induced the change of TRPV1 signal protein from peripheral and central neurons, and that results in visceral hyperalgesia.

IBS has been regarded as the second leading cause of absenteeism only to the common cold [12]. Abdominal pain is a common symptom and difficult to manage effectively in IBS patients. Visceral hyperalgesia has been confirmed as an important factor underlying abdominal pain in IBS patients and could induce exaggerated signal from the gut to the brain. In our study, compared with the control group, the EMG activities of the WAS group were obviously increased when the expansion volume reached 0.2 mL and 0.3 mL. We found that rifaximin could significantly reduce the EMG activities triggered by stress. Our study also showed that there were no significant differences in EMG activities when the distension volume reach 0.2 mL (sham WAS vs. control group: 81.37 ± 4.27 vs. 80.95 ± 4.48, *P* = 0.955) and 0.3 mL (sham WAS vs. control group: 107.62 ± 4.17 vs. 107.19 ± 4.53, *P* = 0.954). However, the obvious differences in EMG activities were observed between the sham WAS group and WAS group. In addition, there were significant differences in EMG activities between the saline+WAS group and Rif+WAS group. The significant differences of EMG activities were not observed among groups when the distension volume exceeds 0.4 mL. These results indicated that the pain threshold of rats with visceral hyperalgesia was reduced, even if mild stimulation could trigger visceral pain sensory in WAS rats. Therefore, we chose slightest stimulation (0.2 mL) to dilate the colorectal in the FMT experiment. In addition, rifaximin could reduce the visceral pain threshold in rats induced by WAS.

The intestinal flora could perform various functions in the human body. It has been proved that probiotic bacteria could relieve the abdominal pain in IBS patients [13, 14], but the exact mechanism was unclear. Recently, many studies have showed that intestinal flora has an important role in the regulation of the gut function, and that intestinal flora was different significantly between IBS patients and healthy subjects by a novel technical method [15]. The composition of intestinal flora was different in fecal from IBS patients by global and deep molecular analysis. Rifaximin has been approved by FDA for the therapy of IBS and was very effective in reducing the symptom of abdominal pain [16, 17]. In our animal studies, compared with the control group, the relative abundance of intestinal flora significantly declines in rats induced by stress. However, rifaximin administration could improve the relative abundance of intestinal flora in rats induced by stress. Rifaximin may improve visceral hypersensitivity by regulating the composition of Spirochaetia and Rikenellaceae in different levels. Intestinal spirochaetosis is located on the luminal surface of colorectal mucosa of animals, which is related to watery diarrhea, abdominal pain, malnutrition, and poor growth. Metronidazole was an effective drug for the treatment of anaerobic spirochaete bacteria. The spirochaete microorganism has been widely reported as existing in the colorectum. If symptomatic, intestinal spirochaetosis commonly presents with watery diarrhea and abdominal pain [18]. One study aimed at evaluating the relationship of intestinal microbiota with stool consistency, psychological distress, and extraintestinal pain in IBS patients, which indicated that lower extraintestinal pain was associated with increased Rikenellaceae [19]. Chronic psychological stress contributes significantly to symptoms of functional bowel disorders and also induces visceral hyperalgesia [20]. The imbalance of intestinal flora may result from psychological stress in animal models of visceral hyperalgesia [21]. Researches showed that the imbalance of intestinal flora, intestinal inflammation, and permeability could be improved by rifaximin in chronic stress rats [3]. We confirmed that stress caused a significant loss of several intestinal flora in different levels. We also showed that oral administration of rifaximin could modulate relative abundance of intestinal flora in different levels.

A study indicated that colonic afferent fibre responses to CRD decreased in rats which were administrated orally with *Lactobacillus reuteri* [22]. Antibiotic treatment could induce the imbalance of intestinal flora in mice, resulting in visceral hyperalgesia, which was associated with increased colonic inflammation and substance P [23]. A study also indicated that bacteria could trigger calcium flux and action potential in nociceptor neurons through different mechanisms [24]. *Lactobacillus reuteri* (DSM 17938) could reduce the jejunal spinal nerve action potentials triggered by capsaicin and distension, and this response was largely blocked by TRPV1 channel antagonist 6-iodonordihydrocapsaicin or in TRPV1 knockout mice [7]. Data indicated that TRPV1 was regarded as a target of neomycin, and the visceral hyperalgesia could be inhibited by intraperitoneal neomycin [25]. Our studies found that rifaximin administration could prevent the increase of TRPV1 in rats induced by stress. We also found that nontoxic stimulus triggered visceral hyperalgesia in rats received FMT from WAS+Rifaximin donor, which was accompanied by changes in TRPV1. Therefore, we speculated that rifaximin improves visceral hyperalgesia via TRPV1 channels by modulating special intestinal flora in rats.

## Data Availability

The raw data supporting the conclusions of this manuscript will be made available by the authors, without undue reservation, to any qualified researcher.
